# Immunostimulatory functions of adoptively transferred MDSCs in experimental blunt chest trauma

**DOI:** 10.1038/s41598-019-44419-5

**Published:** 2019-05-29

**Authors:** Monika Kustermann, Malena Klingspor, Markus Huber-Lang, Klaus-Michael Debatin, Gudrun Strauss

**Affiliations:** 1 Department of Pediatrics and Adolescent Medicine, University Medical Center Ulm, Ulm, Germany; 2 Institute of Clinical and Experimental Trauma-Immunology, University Medical Center Ulm, Ulm, Germany

**Keywords:** Immunology, Cell biology, Immunology, Cell biology

## Abstract

Myeloid-derived suppressor cells (MDSCs) expand during inflammation and exhibit immunomodulatory functions on innate and adaptive immunity. However, their impact on trauma-induced immune responses, characterized by an early pro-inflammatory phase and dysregulated adaptive immunity involving lymphocyte apoptosis, exhaustion and unresponsiveness is less clear. Therefore, we adoptively transferred *in vitro*-generated MDSCs shortly before experimental blunt chest trauma (TxT). MDSCs preferentially homed into spleen and liver, but were undetectable in the injured lung, although pro-inflammatory mediators transiently increased in the bronchoalveolar lavage (BAL). Surprisingly, MDSC treatment strongly increased splenocyte numbers, however, without altering the percentage of splenic leukocyte populations. T cells of MDSC-treated TxT mice exhibited an activated phenotype characterized by expression of activation markers and elevated proliferative capacity *in vitro*, which was not accompanied by up-regulated exhaustion markers or unresponsiveness towards *in vitro* activation. Most importantly, also T cell expansion after staphylococcal enterotoxin B (SEB) stimulation *in vivo* was unchanged between MDSC-treated or untreated mice. After MDSC transfer, T cells preferentially exhibited a Th1 phenotype, a prerequisite to circumvent post-traumatic infectious complications. Our findings reveal a totally unexpected immunostimulatory role of adoptively transferred MDSCs in TxT and might offer options to interfere with post-traumatic malfunction of the adaptive immune response.

## Introduction

Myeloid-derived suppressor cells (MDSCs) are defined as immature myeloid cells with potent immunoregulatory activity whose differentiation is favoured in cancer and other pathological conditions associated with chronic inflammation. MDSC induction, expansion and activation is induced by various factors produced by tumours or bone marrow (BM) stroma in response to chronic inflammation such as IL-6, GM-CSF, G-CSF or VEGF in combination with pro-inflammatory molecules released by activated T cells and myeloid cells or bacterial and viral danger signals as IFN-γ, IL-1β, IL-13 and TLR-ligands^1^. Murine MDSCs consist of two major populations: granulocytic MDSCs (gMDSC) identified by a CD11b^+^Ly-6G^+^Ly-6C^low^ phenotype and monocytic MDSCs (mMDSCs), which are CD11b^+^Ly-6G^−^Ly-6C^high^. Although T cells are the preferred and major targets of MDSC action they modulate the function of other immune cells such as B-, NK- and dendritic cells. Immunosuppressive factors produced by MDSCs are versatile and comprise enzymes catabolizing amino acids essential for T cell activation, proliferation and function such as arginase-1, iNOS and indoleamine 2,3-dioxygenase (IDO), immunosuppressive cytokines like IL-10 and TGF-β, the production of ROS as well as the expression of membrane molecules leading to T cell exhaustion and apoptosis^2–4^. Beside the ability to inhibit expansion and functions of T cells, MDSCs influence the type of immune response by modulating the Th1/Th2 balance depending on the disease entity. While a shift towards type 2 immunity is reported after bone marrow and solid organ transplantation, viral infections, pregnancy or certain cancers, MDSCs promote Th1 responses in airway inflammation^5–11^.

Most of the knowledge regarding the immunosuppressive role of MDSCs has come from studies in the context of cancer. MDSCs in cancer patients and tumour bearing mice strongly suppress the adaptive immune response and favour tumour growth^12,13^. However, their role in other pathological conditions is less well understood. After traumatic insults danger signals activate the innate immune system leading to a local and systemic pro-inflammatory response, which is counterbalanced by a systemic posttraumatic immune suppression characterized by an impaired adaptive immunity leading to a strongly increased risk for opportunistic infections and multi organ failure^14,15^. Due to the post-traumatic inflammatory environment, MDSC accumulation occurs after various traumatic injuries such as spinal cord injury, blunt chest trauma (TxT), sepsis or peripheral tissue trauma. Isolated MDSCs from traumatized animals up-regulate immunosuppressive enzymes such as arginase-1 and iNOS and decrease T cell proliferation *in vitro*^16–21^. The *in vivo* action of trauma-induced MDSCs is less clear and apparently model dependent. Blocking MDSC expansion by anti-Gr-1 antibody in experimental sepsis prevents Th2 polarization of the immune response^20^, while MDSC depletion by anti-Gr-1 inhibits Th1-specific cytokine production without affecting Th2 cytokines in a blunt chest trauma model^19^.

Studying the role of MDSCs *in vivo* is limited by the lack of antibodies specifically deleting MDSCs. The Gr-1 antibody fails to deplete all MDSC subsets in all organs and also eliminates mature neutrophils^19,22–24^. Alternatively, MDSCs at high numbers, quality and purity can be generated *in vitro* and utilized for adoptive transfer experiments. *In vitro*-generated MDSCs are mostly generated from bone marrow (BM) cells, which are cultured for a short period in GM-CSF/G-CSF or M-CSF, often combined with additional factors such as IL-6, TNF α or dexamethasone^5,25–27^. *In vitro*-generated MDSCs exhibit an increased expression of immunosuppressive molecules and efficiently suppress T cell proliferation *in vitro*. Adoptive transfer of *in vitro*-generated MDSCs in experimental models of allogeneic bone marrow or solid organ transplantation promote immune tolerance by preventing lethal graft-versus-host disease (GVHD) and allograft rejection^5,25–27^. Of note, transplantation of *in vitro*-generated MDSCs in a model of spinal cord injury at the side of injury reduce local inflammation and promote tissue regeneration^17^, while the effects of systemically applied MDSCs on the trauma immune response are largely unknown. Therefore, we adoptively transferred *in vitro*-generated MDSCs and analyzed the immune modulating effects on the innate and adaptive immune system in a clinically relevant model of blunt chest trauma.

## Results

### *In vitro*-generated MDSCs home preferentially in the spleen of TxT mice

**Figure 1:**
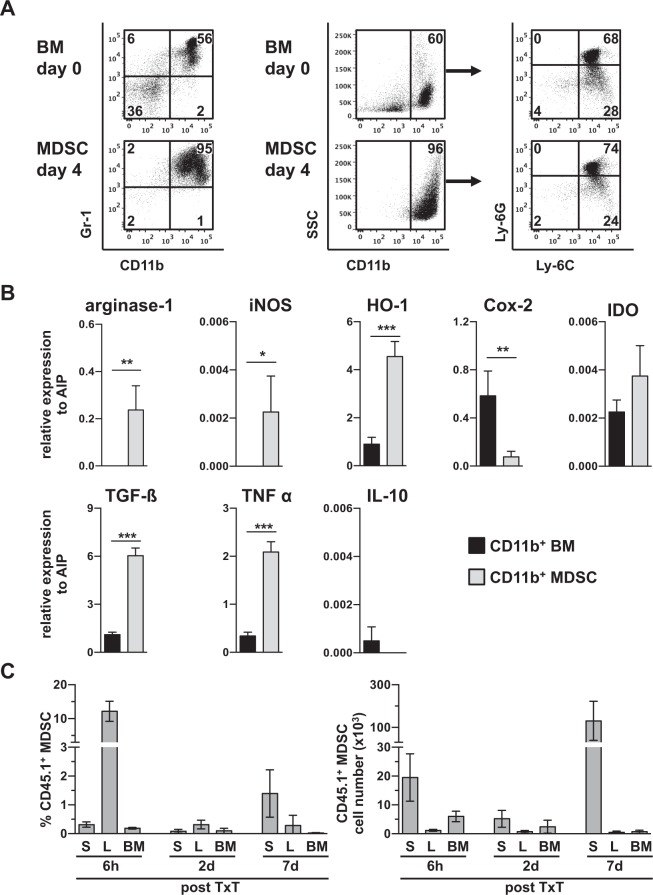
Generation, characterization and homing of adoptively transferred *in vitro*-generated MDSCs. MDSCs were generated *in vitro* from BM cells of B6.SJL mice (H-2^b^, CD45.1^+^) by incubation with GM-CSF. (**A**) After four days cells were stained for CD11b and Gr-1 expression and the expression of Ly-6C and Ly-6G on the CD11b positive cells indicated the distribution of the MDSC subpopulations. (**B**) MDSCs were analyzed for the expression of immunosuppressive mediators by qRT-PCR and relative expression to AIP was calculated. (**C**) MDSCs were injected one hour before TxT in B6 mice (H-2^b^, CD45.2^+^) and homing was analyzed in spleen (S), liver (L) and bone marrow (BM) by analyzing the percentage of CD45.1 expressing cells and subsequently numbers of MDSCs were calculated. Data show one representative FACS staining from 5 independent experiments performed (**A**). (**B**) shows the mean value ± SD of 4 different MDSCs preparations analyzed and (**C**) of 5–11 mice/group analyzed. *P≤0.05; **P≤0.01; ***P≤0.001. Significance was calculated by Student’s t test.

MDSCs were generated from BM cells of B6.SJL mice in the presence of GM-CSF. After four days more than 90% of the cells expressed CD11b and Gr-1, of which granulocytic MDSCs (CD11b^+^Ly-6G^+^Ly-6C^low^) were induced at higher percentages as monocytic MDSCs (CD11b^+^Ly-6G^-^Ly-6C^high^) (Fig. 1A). Compared to CD11b^+^ cells isolated from BM, *in vitro*-generated MDSCs exhibited increased expression of arginase-1, iNOS, HO-1, TGF-β and TNF α, while IDO and IL-10 levels were unchanged and Cox-2 expression was even lower than in BM-isolated CD11b^+^ cells (Fig. 1B). MDSCs efficiently inhibited the proliferation of CD4^+^ and CD8^+^ T cells and suppression slightly varied with each preparation but usually ranged between 60–80% at a MDSC: T cell ratio of 1:1 (Suppl. Fig. S1).

To clarify *in vivo* survival and homing, MDSCs were injected one hour before B6 mice underwent TxT. Due to the congenic marker CD45.1 expressed by B6.SJL mice, transplanted CD45.1^+^ MDSCs could be identified in syngeneic CD45.2^+^ B6 mice. 6 hours after TxT, adoptively transferred MDSCs are predominantly found in the liver, accounting for 12% of liver leukocytes and to a lesser extent in spleen and bone marrow. Two days after injection, MDSCs account for less than 0.1% of total bone marrow and splenocytes and 0.3% of liver leukocytes, while at day seven MDSCs compose 1.4% of splenocytes accounting for 1.3 × 10^5^ MDSCs (Fig. 1C). Most interestingly, MDSCs were not attracted into the injured lung and not detectable in lymph nodes at any time analyzed. These results show, that low numbers of MDSCs preferentially populate the spleen and survive *in vivo* at least seven days after transfer.

### The early TxT-induced local and systemic pro-inflammatory immune response is not substantially modulated by adoptively transferred *in vitro*-generated MDSCs

**Figure 2:**
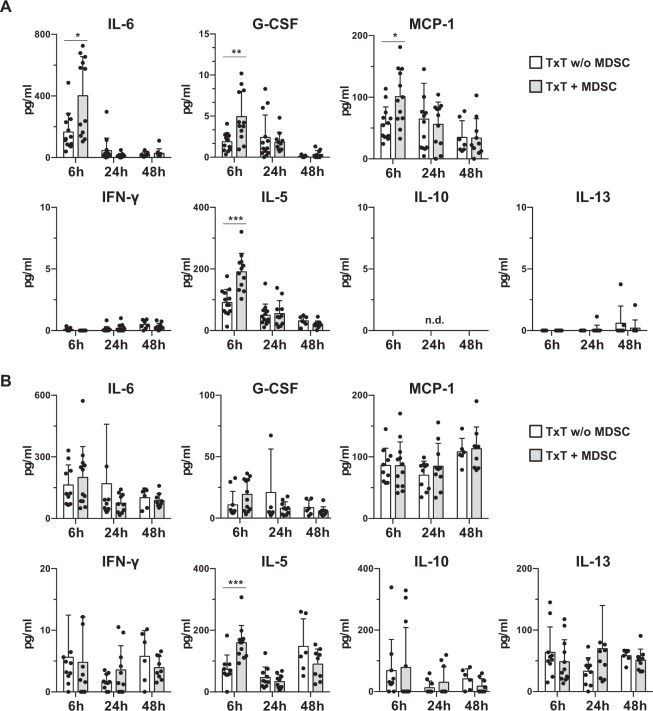
Adoptive transfer of *in vitro*-generated MDSCs in TxT mice does not substantially modulate the expression of cytokines in BAL and serum. TxT mice were treated with MDSCs or left untreated and BAL fluids (**A**) and serum (**B**) were analyzed for cytokine concentrations 6, 24 and 48 hours after TxT. Data present the mean value ± SD of 6–13 mice/group analyzed. *P≤0.05; **P≤0.01; ***P≤0.001. Significance was calculated by Mann-Whitney-U-Test comparing TxT w/o MDSC with TxT + MDSC at each time point.

Since MDSCs are attracted and activated by pro-inflammatory cytokines and chemokines and likewise MDSCs release soluble factors to exert their immunosuppressive function, we analyzed the expression of cytokines and chemokines in the bronchoalveolar lavage (BAL) (Fig. 2A) and the serum (Fig. 2B) of TxT mice treated or untreated with MDSCs 6, 24 and 48 hours after TxT. Pro-inflammatory factors described to be induced shortly after TxT such as IL-6, G-CSF or MCP-1 were slightly increased 6 hours after TxT in MDSC-treated mice in the BAL. Possibly, these factors are derived from the transplanted MDSCs, since *in vitro*-generated MDSCs produce elevated levels of IL-6, G-CSF or MCP-1 compared to CD11b cells isolated from BM (Suppl. Fig. S2), although no invading MDSCs could be detected in the lungs 6 hours after injection by flow cytometry. Already 24 h after TxT, concentrations decreased to levels measured in untreated TxT mice. No additional systemic increase of IL-6, G-CSF and MCP-1 was detectable by MDSCs after TxT. Since MDSCs influence the type of immune response, we determined the concentration of Th1/Th2 specific cytokines. In BAL and serum the concentration of the Th2-specific cytokine IL-5 was increased in MDSC-treated mice 6 hours after TxT, but unaltered compared to untreated animals at later time points. *In vitro*-generated MDSCs, however, do not produce IL-5 (Suppl. Fig. S2). Concentrations of all other Th1- and Th2-specific cytokines analyzed (IFN-γ, IL-10, -13) were not affected by MDSC treatment. All together, these data show that adoptively transferred MDSCs transiently effect cytokine and chemokine levels especially in BAL fluid but do not exhibit long-term effects neither locally in the injured organ nor systemically.

### Adoptive transfer of *in vitro*-generated MDSCs increases splenic leukocyte numbers

**Figure 3:**
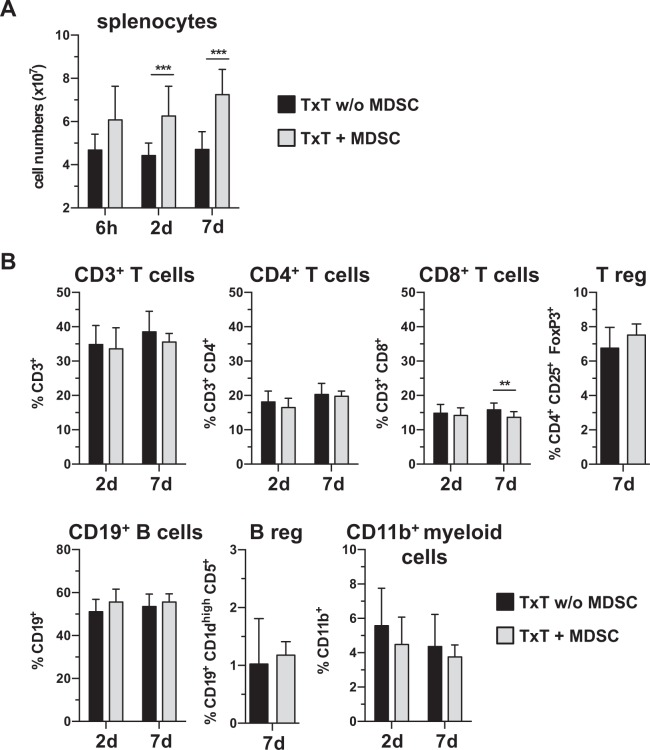
MDSC treatment increases the number of splenic leukocytes, but does not alter the distribution of the different leukocyte subpopulations. MDSC-treated or untreated mice were subjected to TxT. (**A**) Numbers of splenocytes were determined at different time points. (**B**) The distribution of the different leukocyte subsets was analyzed by flow cytometry. Data represent the mean value ± SD for 5–15 mice/group analyzed. **P≤0.01; ***P≤0.001. Significance was calculated by Student’s t-test comparing TxT w/o MDSC with TxT + MDSC for each time point.

Although cytokine and chemokine levels were hardly influenced by MDSC transfer, a massive increase in spleen cell numbers was observed. Two days after adoptive MDSC transfer and TxT, splenocyte numbers were 1.4 fold elevated and increase was maintained after seven days (Fig. 3A). Transplanted MDSCs did not significantly contribute to elevated spleen cell numbers as indicated in Fig. 1C. Although splenic cell numbers increased, the distribution of the different leukocyte subsets (CD3^+^, CD4^+^, CD8^+^, Treg (CD4^+^ CD25^+^ FoxP3^+^), CD19^+^, Breg (CD19^+^ CD1d^high^ CD5^+^) and CD11b^+^) analyzed was unchanged two and seven days after MDSC-treatment (Fig. 3B). Due to elevated splenocyte numbers, total cell numbers of the different cell populations were increased in MDSC-treated mice compared to untreated controls (Suppl. Fig. S3A). To clarify, whether MDSC-induced expansion of splenocytes is specific for the “traumatic environment”, we injected MDSC in untreated B6 mice. Two and seven days after MDSC injection no increase in splenocyte numbers was detected (Suppl. Fig. S3B). In summary, these results show that treatment of TxT mice with *in vitro*-generated MDSCs induces splenic leukocyte expansion.

### Adoptive transfer of *in vitro*-generated MDSCs induces T cell activation without induction of exhaustion

**Figure 4:**
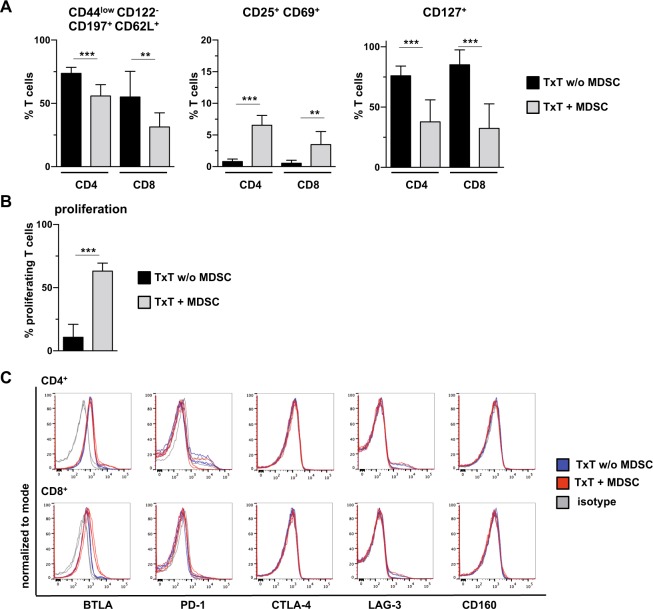
Adoptive transfer of *in vitro*-generated MDSCs activates TxT-induced T cells without inducing exhaustion. Mice were untreated or adoptively transferred with MDSCs and subsequently received TxT. (**A**) 7 days later, splenocytes were cultured for 24 h and CD4^+^ and CD8^+^ T cells were stained for markers defining naïve and activated cells. (**B**) At day 7 after TxT, CFSE-labelled splenocytes were cultured in medium and 4 days later proliferation of CD3^+^ T cells was analyzed. (**C**) 7 days after TxT, splenocytes were cultured for 24 h and expression of exhaustion markers was defined on CD4^+^ and CD8^+^ T cells. **P≤0.01, ***P≤0.001 Data represent the mean value ± SD of 8–10 animals/group (**A**) and 13–14 mice/group (**B**). (**C**) FACS staining’s show the expression of markers for 3 mice/group and is representative for one experiment out of 2 experiments performed. Significance was calculated by Student’s t-test.

Since adoptive transfer of MDSCs induced expansion of T cells, which represent the most prominent targets of MDSC action, we defined the impact of MDSCs on T cell activation. Seven days after MDSC injection and TxT, spleen cells were stained after 24 hours culture for activation markers. Most of the T cells independent whether they were derived from MDSC-treated or untreated TxT mice were naïve T cells (CD44^low^ CD122^-^ CCR7^+^ (CD197) CD62L^+^), although a decrease from 74% to 56% for CD4^+^ and from 55% to 32% for CD8^+^ T cells was observed in MDSC-treated mice. Likewise about 7% of the CD4^+^ T cells and 4% of the CD8^+^ T cells co-expressed the activation markers CD25 and CD69 and T cells showed diminished IL-7 R (CD127) expression indicating that the presence of MDSCs apparently sensitized T cells towards an activated state (Fig. 4A). This was further confirmed by the finding that CFSE-labelled splenic T cells isolated from MDSC-treated TxT mice after 7 days start to proliferate when cultured in medium for 4 days (Fig. 4B). While CD3^+^ T cells from untreated TxT mice showed a spontaneous proliferation of 11%, 63% of T cells from MDSC-treated TxT mice divided in culture. Since persistent activation is often associated with T cell exhaustion, expression of exhaustion markers was defined 24 hours after culture. Only BTLA (CD272) was expressed on CD4^+^ and CD8^+^ T cells, however, independent of MDSC-treatment. All other analyzed exhaustion markers PD-1 (CD279), CTLA-4 (CD152), LAG-3 (CD223) and CD160 were not up-regulated (Fig. 4C). In summary, the presence of adoptively transferred MDSCs activates T cells without driving them into exhaustion.

### Adoptive transfer of *in vitro*-generated MDSCs in TxT mice does not impair the proliferative capacity of T cells *in vitro* and *in vivo* after activation

**Figure 5:**
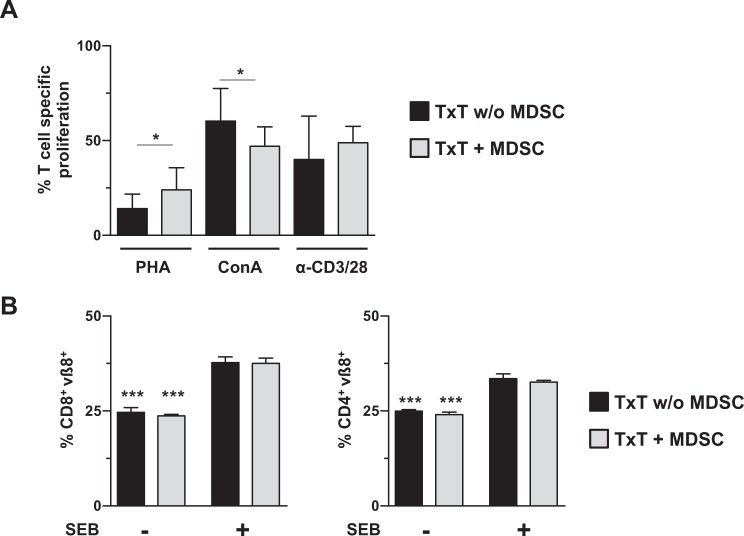
T cells from MDSC-treated mice maintain their proliferative capacity *in vitro* and *in vivo*. MDSC-treated or untreated mice were subjected to TxT. (**A**) 7 days after TxT, spleen cells of MDSC-treated or untreated TxT mice were CFSE-labelled and stimulated with medium, PHA, ConA or anti-CD3/28 antibodies and after 4 days proliferation of CD3^+^ T cells was analyzed and percentage of specific proliferation was calculated. (**B**) 24 h after TxT, mice were injected with SEB and the percentage of proliferating vß8^+^ CD4^+^ and vß8^+^ CD8^+^ T cells was defined 48 hours later by flow cytometry. *P≤0.05; ***P≤0.001. Data represent the mean value ± SD of 13–14 mice/group (**A**) and 3–4 mice/group (**B**). Significance was calculated by Student’s t-test comparing TxT w/o MDSC with TxT + MDSC for each stimulus (**A**) and by one-way ANOVA with Sidak as post test (**B**).

Since T cells from TxT mice received an activation signal by adoptively transferred MDSCs, we next clarified whether T cells maintained their ability to proliferate after activation. Seven days after TxT, spleen cells from MDSC-treated or untreated mice were activated by PHA, ConA or anti-CD3/CD28 antibodies. Surprisingly, the presence of MDSCs in TxT mice did not strongly modulate the proliferative capacity of CD3^+^ T cells. While PHA-induced proliferation was slightly increased, the response towards ConA was faintly attenuated and no difference was detected for anti-CD3/CD28 activation (Fig. 5A). To clarify, whether MDSC-treated TxT mice can respond to T cell activation *in vivo*, staphylococcal enterotoxin B (SEB), which specifically induces expansion of T cells bearing vβ8 TCRs^28^, was injected 24 hours after TxT. MDSC-treatment in the absence of SEB had no impact on the percentage of vβ8 expressing T cells, and SEB injection induced the proliferation of CD4^+^ and CD8^+^ vβ8^+^ T cells, however, to the same extend in MDSC-treated and untreated animals (Fig. 5B). All together, these results show that adoptively transferred *in vitro*-generated MDSCs in the context of TxT do not inhibit the proliferative capacity of T cells *in vitro* and *in vivo*.

### T cells from TxT mice maintain a Th1 phenotype in the presence of *in-vitro* generated MDSCs

**Figure 6:**
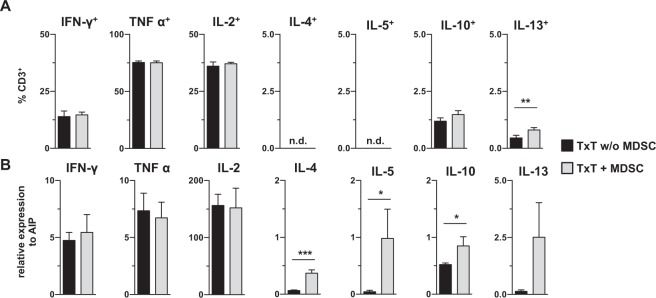
The adoptive transfer of *in vitro*-generated MDSCs maintains the Th1/Th2 balance in TxT mice. Mice were untreated or adoptively transferred with MDSCs and subsequently received TxT. (**A**) 7 days after TxT, splenocytes were restimulated with PMA/Iono and the intracellular expression of Th1- and Th2-associated cytokines was determined in the CD3^+^ T cell population by flow cytometry. (**B**) Alternatively, after PMA/Iono stimulation, CD3^+^ T cells were isolated and qRT-PCRs for the expression of Th1-and Th2-associated cytokines were performed and relative expression to AIP was calculated. *P≤0.05; **P≤0.01; ***P≤0.001. Data represent the mean value ± SD for 3–4 mice/group in (**A**) and (**B**) and significance was calculated by Student’s t-test comparing TxT w/o MDSC with TxT + MDSC for each cytokine.

Since post-traumatic immunosuppression is associated with a shift in the immune response towards type 2 immunity^29,30^ and MDSCs are modulators of the Th1/Th2 balance, we analyzed the polarization of T cells after adoptive transfer of MDSCs. MDSC-treated or untreated mice received TxT and seven days later spleen cells were restimulated with PMA/ionomycin, and cytokine expression of CD3^+^ T cells was analyzed by intracellular flow cytometry (Fig. 6A). Most of the T cells exhibit a Th1 phenotype. Less than 2% of T cells expressed IL-10 and IL-13, while IL-13 expressing cells were slightly increased in MDSC-treated mice. Since IL-4 and IL-5 expressing cells were not detectable by flow cytometry, mRNA expression in isolated splenic T cells of MDSC-treated and untreated mice after PMA/ionomycin stimulation was analyzed. Independent of the presence or absence of MDSCs, Th1-associated cytokines were strongly expressed, while Th2-associated cytokine expression was low. However, T cells from MDSC-treated mice exhibited higher expression of IL-4, -5, -10 and -13 (Fig. 6B). In summary, adoptive transfer of MDSCs in TxT mice does not significantly shift the Th1/Th2 balance but slightly promote the induction of Th2 cells.

## Discussion

Severe trauma does not only affect the function of the injured organ but is associated with profound changes in immunohomeostasis characterized by an imbalanced and overwhelming innate immune response and a severe maladaptive immunity. MDSCs are induced after various experimental traumatic injuries due to systemic inflammation; however, their immunomodulatory effects on the subsequent immune response are less well defined. To our knowledge, we show here for the first time that the adoptive transfer of *in vitro*-generated MDSCs shortly before experimental TxT induces the expansion and activation of T cells without dampening their proliferative capacity, inducing exhaustion or influencing the Th1/Th2 balance. These results indicate that in the context of TxT, adoptively transferred MDSCs do not exhibit classical immunosuppressive functions but rather act immunostimulatory.

Recently, we showed that MDSCs are induced locally in the lung and systemically in the spleen shortly after TxT. TxT-induced MDSCs inhibited T cell proliferation when tested for their immunosuppressive capacity *in vitro*. By treating mice with the anti-Gr-1 antibody, which is commonly used for MDSC depletion, we found that the presence of MDSCs dampened the early systemic pro-inflammatory response and guaranteed the induction of Th1 cells^19^. Since, anti-Gr-1 treatment does not exclusively and entirely deplete all MDSCs^19,23,24^, adoptive transfer of *in vitro*-generated MDSCs offers an attractive alternative to define their influence on disease-induced immune responses and finally their therapeutic potential. In contrast to experimental models of autoimmunity or solid and BM transplantations, not much is known about the effect of *in vitro*-generated MDSCs on the course of traumatic injuries and their influence on the trauma-induced immune response. *In vitro*-generated MDSCs applied intraspinally after spinal cord injury reduce inflammation and promote tissue generation^17^. Furthermore, the adoptive transfer of MDSCs exhibit a cardioprotective role in heart failure^31^ and immature myeloid cells resembling MDSCs enhance bone fracture healing^32^.

Therefore, we adoptively transferred *in vitro*-generated MDSCs one hour before mice received TxT and analyzed changes in innate and adaptive immune responses. GM-CSF-induced MDSCs derived from BM cells exhibited increased expression of arginase-1, iNOS, HO-1, TGF-β and TNF-α and efficiently suppressed allogeneic-induced T cell proliferation *in vitro*. Adoptively transferred MDSCs homed preferentially in the spleen, but low numbers of MDSCs were also found in the liver and bone marrow. Although no MDSC infiltration into the injured lung was detected, pro-inflammatory mediators typically elevated in the BAL after TxT were further increased six hours after MDSC treatment while at later time points MDSC did not affect cytokine and chemokine expression. Likewise, no systemic effect was detectable indicating that the post-traumatic early inflammatory response cannot be efficiently modulated by adoptively transferred MDSCs.

Interestingly, the adoptive transfer of MDSCs strongly increased total splenocyte numbers particular in TxT mice, since non-traumatized mice did not exhibit elevated cell numbers pointing to an influence of the TxT microenvironment on MDSC functions. Since the ratio of the different splenic leukocyte subpopulations was unaltered, total cell numbers of all subpopulations analyzed were increased. B cells with a regulatory phenotype are expanded after adoptive transfer of *in vitro*-generated MDSCs in a murine model of systemic lupus erythematosus (SLE) and experimental autoimmune encephalomyelitis, however, MDSC treatment in this model simultaneously decreases the numbers of activated T cells^33,34^. Similarly, Tregs are reported to be induced after MDSC transfer dependent on the model used. While adoptive transfer of MDSCs in mice with collagen-induced arthritis or SLE show no Treg induction^35,36^, Treg frequency is increased for instance in experimental asthma or viral myocarditis models^37,38^.

Increase of all splenic leucocyte populations after MDSC transfer is to our knowledge not described yet, however, several studies indicate that MDSCs exhibit alternative functions in place of immunosuppression. MDSCs induced by inflammatory bowel disease in mice and patients not only fail to suppress autologous T cell responses but even enhance T cell proliferation *in vitro*^39^. Adoptive transfer of MDSCs in skin-transplanted mice strongly increases the numbers of infiltrating T cells in the transplant and induces the expansion of APCs and activated CD25^+^ CD69^+^ T cells in the spleen pointing to a state of systemic activation, which, surprisingly, does not lead to transplant rejection but correlates with prolonged skin graft survival^40^. Immunostimulatory capacities are also described for ascites-derived CD11b^+^ Gr-1^+^ leading to enhanced T cell proliferation *in vitro* and *in vivo*^41^. Likewise, T cells conditioned *in vitro* with MDSCs show an increased anti-tumour activity after adoptive T cell based immunotherapy, which is associated with increased IFN-γ expression and diminished mTOR signalling^42^.

The above reported immunoactivating functions of MDSCs in models of autoimmunity, cancer and transplantation correspond to our findings in the TxT model. After MDSC-treatment T cells from TxT mice showed elevated expression of activation markers and increased proliferative capacity in the absence of activation signals. However, these intrinsic potential to proliferate does not impair the ability to respond to appropriate activation signals *in vitro* and *in vivo* and most interestingly was not associated with T cell exhaustion, although *in vitro*-generated MDSCs exhibit high expression of PD-L1.

Beside their impact on T cell activation and expansion, MDSCs strongly modulate the type of T cell response induced. Dysregulation of the Th1/Th2 balance frequently occurs after severe traumatic injuries and suppression of Th1 responses associated with enhanced Th2 immunity contribute to the predisposition of injured individuals for infections, sepsis and impaired pathogen defence^29,30,43,44^. Although adoptively transferred MDSCs exhibited a strong effect on T cell expansion and activation, their influence on T cell polarization was marginal. Th2-associated cytokines were expressed at low levels after TxT and slightly increased in the presence of MDSCs, but the predominance of Th1 immunity required to resolve post-traumatic infections was maintained.

Our studies clearly indicate a discrepancy between the *in vitro* and *in vivo* action of MDSCs. While *in vitro*-generated MDSCs strongly prevent T cell proliferation *in vitro*, they induce T cell expansion *in vivo*. Studies by Schmidt *et al*. could also show that tumour-induced MDSCs efficiently suppress CTLs *in vitro*, but not *in vivo* following adoptive transfer^45^. This strongly implies that the microenvironment, immune response and MDSC functions form an intricately interwoven network of mutual influences. MDSCs isolated from late septic mice and subsequently transferred to septic mice decrease pro-inflammatory cytokines, increase bacterial clearance and dramatically improve survival rates. If MDSCs, however, are isolated from early septic mice representing an altered inflammatory environment and subsequently transferred into septic mice they than support disease progression^46^.

Adoptive transfer of *in vitro*-generated MDSCs in allogeneic BM transplantation models inhibit GVHD by inducing type 2 immunity without affecting T cell numbers^5^, while MDSCs generated in the exactly same manner induced lymphocyte expansion and T cell activation without influencing the Th1/Th2 balance in the TxT model. Likewise, the transfer of MDSCs in models of asthma-related airway inflammation diminished the inflammatory injury by shifting the balance towards a Th1 response^11,47^. Most interestingly, this is independent, whether MDSCs were derived from mice treated with LPS or isolated from BM of tumour bearing mice, although MDSCs in the context of cancer are known to promote Th2 responses^9,10,48^. Depending on the disease model in which MDSCs are induced or on the time point they are isolated, MDSCs exhibit versatile mechanisms of immunomodulation comprising the release of soluble factors, the induction of other regulatory cell types and the direct interaction with their target cells.

How MDSCs induce the induction and activation of lymphocytes in TxT is currently unclear. Comparing the transcriptome, proteome and secretome of MDSCs isolated from models such as BM transplantation and TxT, in which MDSCs exhibit totally opposite immunomodulatory effects, might depict candidates responsible for immunoactivating or immunosuppressing functions of MDSCs.

Further studies have to elucidate whether the immunostimulatory effect of adoptively transferred MDSCs is found also in other trauma patterns and most importantly, whether similar changes in the immune response are observed when MDSCs are injected after the traumatic injury, especially in order to evaluate their therapeutic potential to improve the post-traumatic immunohomeostasis. This might be of particular relevance for diseases such as sepsis, often manifested by severe, long-term maladaptive immune responses and immunosuppression associated with lymphocyte exhaustion and apoptosis, a failure to return to normal immunohomeostasis and inefficient response to secondary infections^49^.

Taken together, we suppose that *in vitro*-generated MDSCs in the context of traumatic injury do not exhibit immunosuppressive activity but activate the T cell response, which might be beneficial to dampen post-traumatic T cell malfunctions. Furthermore, we could show that MDSCs are immunoregulators with a high plasticity which after adoptive transfer acquire regulatory functions ranging from T cell inhibition to T cell activation according to the disease entity and that it is worthwhile and absolutely necessary to define the effect of adoptive MDSC transfer in each individual disease model.

## Material and Methods

### Animals and blunt chest trauma (TxT)

All animal experiments were performed according to the international regulations for the care and use of laboratory animals and were approved by the local Ethical Committee (No. 1196 and 1297, Regierungspräsidium Tübingen, Germany). Male C57BL/6 mice (B6, H-2^b^, CD45.2) (Janvier, France) were used for TxT at an age between 11 to 15 weeks. Male B6 and B6.SJL-Ptprc^a^Pepc^b^/BoyJ (B6.SJL, H-2^b^, CD45.1) mice (breeding pairs obtained from The Jackson Laboratory and bred at University of Ulm) were between 6–14 weeks for MDSC generation from bone marrow (BM). TxT was induced by a single blast wave centered on the thorax under sevoflurane anesthesisa as described previously^19^. In brief, compressed air was delivered in the upper chamber of the blast wave generator, which is divided by the lower chamber by a Mylar polyester film. As soon as the pressure in the upper part exceeded the defined resistance of the membrane, the film ruptured towards the nozzle and released a reproducible single blast wave and contusion of the lung, which is not associated with histological alterations in liver or abdomen. The sternum cylinder distance was 1.5 cm. Mice received buprenorphin 0.03 mg/kg 30 minutes before TxT and every 8 hours during the first 24 hours after TxT as analgetic treatment.

### Bronchoalveolar lavage (BAL) and blood serum

BAL samples were obtained as described previously^19^. Briefly, trachea was exposed, cannulated and afterwards, 0.5 ml ice-cold PBS was injected and recovered. 1 µl proteinase inhibitor cocktail was added to 100 µl BAL and samples were centrifuged at 13,000 rpm for 1 min. Supernatant fluid was stored at −80 °C. Blood was collected after a puncture of the mandibular vein, incubated at room temperature for 30 min and centrifuged at 13,000 rpm at 4 °C for 15 min. Cytokine stabilization buffer (U-CyTech biosciences) was added to the serum and stored at −80 °C. Cytokine concentrations were determined by Procartaplex Multiplex Immunoassays (Thermo Fisher Scientific) and analyzed on a BIO RAD-Bio-Plex 200 System (Bio-Rad).

### MDSC generation and adoptive transfer of MDSCs

3 × 10^5^ BM cells/ml extracted from the femur and tibia were cultured with granulocyte-macrophage colony-stimulating factor (GM-CSF) (250U/ml) (Peprotech) for 4 days. If more than 90% of the cells expressed CD11b and Gr-1, 2 × 10^7^ MDSCs were adoptively transferred into tail veins of B6 mice one hour before TxT.

### Isolation of cells

#### Magnetic bead isolation

CD3^+^ T cells were isolated from spleens by CD3ε MicroBead Kit and CD11b^+^ cells were isolated from BM by using CD11b MicroBead Kit (Miltenyi). Cells were positively selected by MACS technology according to manufacture’s protocol. Purity of all isolated cells ranged between 85–99%.

Leukocytes of different organs were isolated according to following protocols:

#### Bone marrow

Under sterile conditions, BM was isolated from the femurs and tibias of mice and a single cell suspension was prepared by dissociating cell clumps with a syringe followed by the lysis of erythrocytes (0.15 M NH_4_Cl, 1 mM KHCO_3_, 0.1 mM Na_2_EDTA).

#### Spleen

The spleen was extracted from mice and a single cell suspension was prepared by gently pressing the spleen through a cell strainer (∅ 70 μm) and subsequently, the erythrocytes were lysed.

#### Liver

The liver was perfused with liver perfusion medium (Invitrogen Life Technologies) followed by liver digest medium (Invitrogen Life Technologies) afterwards removed and digested for 30 min at 37 °C. Liver cells were gently pressed trough a cell strainer (∅ 70 µm) and lymphoid cells were separated by centrifugation 60 × g for 5 min. Lymphoid cells in the supernatant were collected, washed and resuspended in PBS supplemented with 1% FCS and diluted in 70% Easycoll (Biochrom) in a 1:1 ratio (≙ 35% Easycoll) and then overlaid onto 70% Easycoll and centrifuged for 20 min at 950 × g. Lymphoid cells were collected from the interface, washed and subsequently erythrocytes were lysed.

### CFSE-labelling and T cell activation *in vitro* and *in vivo*

2 × 10^6^ spleen cells were labelled with 5 µM CFSE (Thermo Fisher Scientific) at 37 °C for 10 min, immediately washed with ice-cold PBS-5%FCS, and subsequently used for proliferation assays. 2.5 × 10^6^/ml CFSE-labelled spleen cells of TxT mice were activated with anti-CD3 (cl. 145–2C11, 0.005 µg/ml) and anti-CD28 (cl. 37.51, 0.005 µg/ml) (CD3/28) antibodies (BD Bioscience), phytohaemagglutinin (PHA, 1.25 µg/ml, Sigma), concanavalin A (Con A, 1.25 µg/ml, Sigma) or incubated with medium and proliferation of CD3^+^ T cells was determined on day 4 by flow cytometry. Percentage of specific proliferation = (% stimulus-induced proliferating T cells - % proliferating T cells in medium alone)/(100 - % proliferating T cells in medium alone) x100. 24 hours after TxT 50 µg staphylococcal enterotoxin B (SEB, Sigma-Aldrich)/mouse or PBS was injected i.v. in the tail vein. Expansion of vß8^+^ T cells was determined 48 h later by flow cytometry.

### Flow cytometry

A total of 5 × 10^5^ cells were stained in FACS-medium (PBS-10% FCS-0.2% NaN_3_). Dead cells were gated out by analyzing 7-amino-actinomycin-D (7-AAD, Sigma-Aldrich) negative cells. For intracellular cytokine detection, spleen cells were restimulated with phorbol myristate acetate (PMA) (20 ng/ml) plus ionomycin (1 µM) (Calbiochem) in the presence of brefeldin A (10 µg/ml) (Sigma-Aldrich). After 5 hours, cells were stained for CD3 expression, fixed with 4% paraformaldehyde, subsequently lysed with 0.1% saponin (Sigma-Aldrich) and stained for cytokine expression. Staining of regulatory T cells (T reg) was performed according to the manufacturer’s instructions by using the Foxp3 Transcription Factor Staining Buffer Kit (Life Technologies). Antibodies used are specified in Supplementary Table 1. Flow cytometry samples were measured on LSR II flow cytometer (BD Bioscience).

### RNA preparation and quantitative reverse-transcription polymerase chain reaction (qRT-PCR)

RNA was isolated and complementary DNA was synthesized as previously described^19^ and qRT-PCR was performed with a CFX Connect™ Real-Time PCR Detection System (Bio-Rad) using a LightCyler FastStart DNA Master PLUS SYBR Green I Kit (Roche Diagnostics). The qRT-PCR results were normalized using mouse aryl hydrocarbon receptor-interacting protein (AIP) as house keeping gene. Primer sets (Thermo Fisher Scientific) used are listed in Supplementary Table 2.

## Statistics

Data were analyzed by either using a Student’s t test, Mann-Whitney-U-test or one-way ANOVA followed by a Sidak test as a post hoc test for multiple comparisons. Results were considered as significant if P≤ 0.05.

## Supplementary information

Dataset 1

## Data Availability

The data sets generated during the current study are available from the corresponding author on reasonable request.
