# Supplementary material for: Immunostimulatory functions of adoptively transferred MDSCs in experimental blunt chest trauma
Source: Sci Rep. 2019 May 29;9:7992. doi: 10.1038/s41598-019-44419-5 (PMC6541619; doi:10.1038/s41598-019-44419-5)

Immunostimulatory functions of adoptively transferred MDSCs in experimental blunt chest trauma

Monika Kustermann[1], Malena Klingspor[1], Markus Huber-Lang[2], Klaus-Michael Debatin[1] and Gudrun Strauss[1]

[1]Department of Pediatrics and Adolescent Medicine, University Medical Center Ulm, Ulm, Germany; [2] Institute of Clinical and Experimental Trauma-Immunology, University Medical Center Ulm, Ulm, Germany

Supplementary Material

Table S1. Antibodies

Epitope	clone	company
CD1d	1B1	Thermo Fisher Scientific
CD3ε	17A2	Thermo Fisher Scientific
CD3	145-2C11	BD Bioscience
CD4	RM4-5	Thermo Fisher Scientific
CD5	53-7.3	Thermo Fisher Scientific
CD8	53-6.7	Thermo Fisher Scientific
CD11b	M1/70	Thermo Fisher Scientific
CD19	1D3	Thermo Fisher Scientific
CD25	PC61.5	Thermo Fisher Scientific
CD28	37.51	BD Bioscience
CD44	IM7	Thermo Fisher Scientific
CD45.1	A20	BD Bioscience
CD62L	MEL-14	BD Bioscience
CD69	H1.2F3	BD Bioscience
CD122	TM-ß1	Thermo Fisher Scientific
CD127	A7R34	Thermo Fisher Scientific
CD152 (CTLA-4)	UC10-4B9	BioLegend
CD160	7H1	BioLegend
CD197	4B12	Thermo Fisher Scientific
CD223 (LAG-3)	C9B7W	Thermo Fisher Scientific
CD272 (BTLA)	6A6	BioLegend
CD279 (PD-1)	RMP1-30	Thermo Fisher Scientific
FoxP3	FJK-16s	Thermo Fisher Scientific
Gr-1	RB6-8C5	Thermo Fisher Scientific
IFN-γ	XMG1.2	Thermo Fisher Scientific
IL-2	JES6-5H4	Thermo Fisher Scientific
IL-4	11B11	Thermo Fisher Scientific
IL-5	TRFK5	Thermo Fisher Scientific
IL-10	JES5-16E3	Thermo Fisher Scientific
IL-13	eBio13A	Thermo Fisher Scientific

Ly-6C	HK1.4	Thermo Fisher Scientific
Ly-6G	1A8	BD Bioscience
TNF α	MP6-XT22	BioLegend
vβ8.1.2.3	F23.1	BD Bioscience

Table S2. qRT-PCR Primer

target	Sequence (5´to 3´)
AIP forward	GCTCCGTTATAGATGACAGC
AIP reverse	ATCTCGATGTGGAAGATGAG
arginase-1 forward	TCCTTTCAAATTGTGAAGAACCCACGGTC
arginase-1 reverse	AGAATCCTGGTACATCTGGGAACTTTCCT
Cox-2 forward	GGCGCAGTTTATGTTGTCTGT
Cox-2 reverse	CAAGACAGATCATAAGCGAGGA
G-CSF forward	TATAAAGGCCCCCTGGAGCTG
G-CSF reverse	GCTGCAGGGCCATTAGCTTC
HO-1 forward	ACAACCAGTGAGTGGAGCCT
HO-1 reverse	TCAAGGCCTCAGACAAATCC
IDO forward	GGATGCGTGACTTTGTGGAC
IDO reverse	TTCTTTGCCAGCCTCGTGT
IL-2 forward	AACCTGAAACTCCCCAGGAT
IL-2 reverse	GTCAAATCCAGAACATGCCG
IL-4 forward	GGTGTTCTTCGTTGCTGTGA
IL-4 reverse	TCTCGAATGTACCAGGAGCC
IL-6 forward	GTCCTTCCTACCCCAATTTCCA
IL-6 reverse	TAACGCACTAGGTTTGCCGA
IL-5 forward	CCCACGGACAGTTTGATTCT
IL-5 reverse	GCAATGAGACGATGAGGCTT
IL-10 forward	CACTGCTATGCTGCCTGCTCTTACTGAC
IL-10 reverse	TGGCAACCCAAGTAACCCTTAAAGTCCT
IL-13 forward	AGGGCCGGTGCCAAGATCTG
IL-13 reverse	GGGAGGCTGGAGACCGTAGTG
iNOS forward	AGCAATGGGCAGACTCTGAAGAAATCTC
iNOS reverse	ATGTTTGCTTCGGACATCAAAGGTCTCAC
MCP-1 forward	TGCCCTAAGGTCTTCAGCAC
MCP-1 reverse	AAGGCATCACAGTCCGAGTC
TGF-ß forward	TCTACCAGAAATATAGCAACAATTCCTGGCGT
TGF-ß reverse	CTGAATCGAAAGCCCTGTATTCCGTCTC
TNF α forward	CCAGACCCTCACACTCAGATCATCTTCTC
TNF α reverse	CTAGTTGGTTGTCTTTGAGATCCATGCCGT

Figure S1. In vitro-generated MDSCs prevent allogeneic T cell proliferation in vitro.

CFSE-labelled B6.SJL spleen cells (H-2^b, CD45.1^+) were activated alloantigen-specifically with irradiated spleen cells of DBA/2 mice (H-2^d, CD45.2^+) in the presence of decreasing numbers of in vitro-generated MDSCs derived from B6 mice (H-2^b, CD45.2^+). After 4 days, cells were stained for CD45.1, CD4 and CD8 and proliferation of CD45.1^+ $CD4^+$ and CD45.1^+ $CD8^+$ T cells was analyzed by CFSE dilution and suppression was calculated. Percentage suppression = 100 – (% proliferating T cells + stimulators + MDSCs) / (% proliferating T cells + stimulators) x 100. Data represent 2 independent experiments.

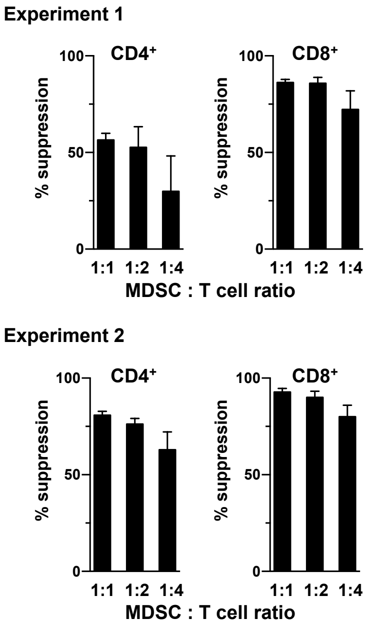

Figure S2. Cytokine expression of in vitro-generated MDSCs.

RNA expression of various cytokines was compared between $CD11b^+$ cells isolated by magnetic beads from bone marrow (BM) cells of B6 mice and in vitro-generated MDSCs by qRT-PCRs and relative expression to AIP was calculated. *P≤0.05; ***P≤0.001. n.d. = not detectable. Data represent the mean value ± SD of RNA isolated from 4 mice/group. Significance was calculated by Student´s t-test.

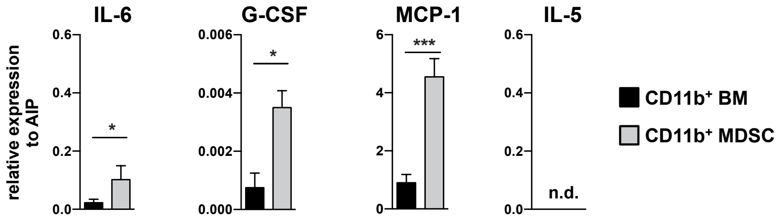

Figure S3. MDSC treatment increases the numbers of splenic leukocytes only in TxT mice.

(A) Splenocytes of MDSC-treated and untreated TxT mice were stained for different leukocyte populations at day 2 and 7 after TxT (T cells: $CD3^+$, $CD4^+$ T cells: $CD3^+CD4^+$, $CD8^+$ T cells: $CD3^+CD8^+$, Treg: $CD4^+CD25^+FoxP3^+$, B cells: $CD19^+$, Breg: $CD19^+CD1d^{high}CD5^+$, myeloid cells: $CD11b^+$) and cell numbers were calculated. (B) B6 mice were adoptively transferred with MDSCs or left untreated and splenic cell numbers were analyzed two or seven days after transfer. *P≤0.05; **P≤0.01; ***P≤0.001. Data represent the mean value ± SD for 5-15 mice/group (A) and 5-7 mice/group (B). Significance was calculated by Student´s t-test comparing TxT w/o MDSC with TxT + MDSC mice (A) or B6 w/o MDSC with B6 + MDSCs (B) for each time point.

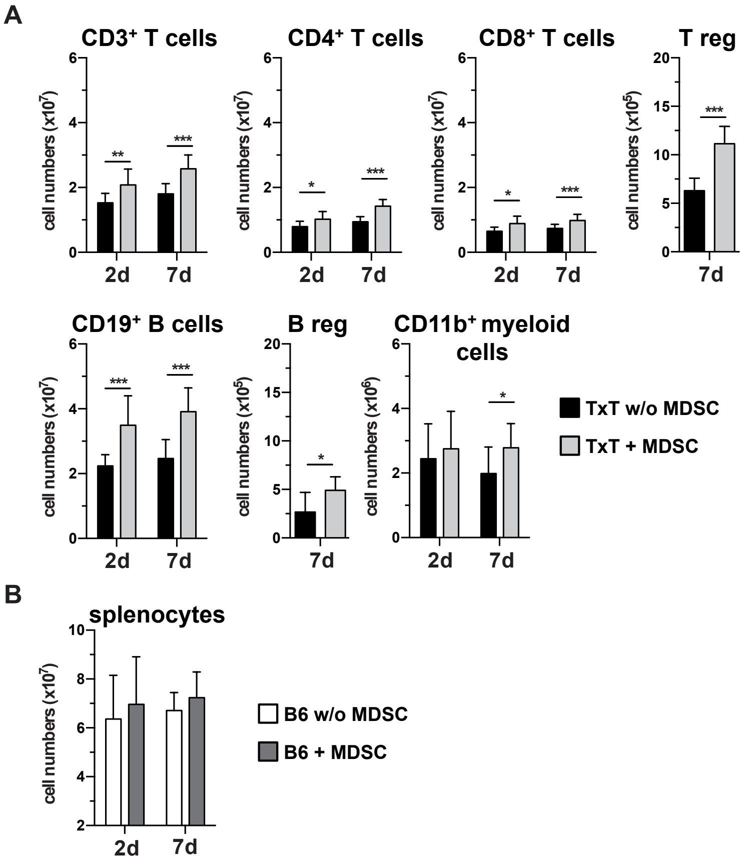

Supplement: Supplementary file 1 — Dataset 1 [file 41598_2019_44419_MOESM1_ESM.pdf]
